# A System for Monitoring Animals Based on Behavioral Information and Internal State Information

**DOI:** 10.3390/ani14020281

**Published:** 2024-01-16

**Authors:** Taro Shibanoki, Yuugo Yamazaki, Hideyuki Tonooka

**Affiliations:** 1 Department of Intelligent Mechanical Systems, Faculty of Environmental, Life, Natural Science and Technology, Okayama University, Okayama 700-8530, Japan; 2 Major in Computer and Information Sciences, Graduate School of Science and Engineering, Ibaraki University, Hitachi 316-8511, Japan; 21nm760a@vc.ibaraki.ac.jp (Y.Y.); hideyuki.tonooka.dr@vc.ibaraki.ac.jp (H.T.)

**Keywords:** monitoring system, image processing, mask R-CNN, anomaly detection, one-class SVM, rodents

## Abstract

**Simple Summary:**

In the current study, we proposed a system that discriminates the state of an animal by combining two types of features: behavioral information, which determines the kind of movement performed by the animal, and internal information, which reflects the animal’s internal state. The proposed system measures biological information from camera images and extracts features to discriminate states using machine learning. In addition, a neural network was used to increase the accuracy of detecting the target, and an anomaly detection method was used to perform discrimination. In the experiments performed, we prepared video images containing routine behaviors and generated a model that can detect non-routine behaviors using the proposed system. We also prepared video images including non-routine behavior in which the hamster was stimulated by clapping to generate an abnormal sound. In the video, the behavior changed significantly before and after the stimulus presentation, and the extracted feature values also changed according to the behavior. As a result of detection, the system discriminated the behavior as non-routine behavior after the stimulation. In conclusion, the results supported the possibility of using a pet monitoring system to immediately inform the owner when the animal is under load.

**Abstract:**

Managing the risk of injury or illness is an important consideration when keeping pets. This risk can be minimized if pets are monitored on a regular basis, but this can be difficult and time-consuming. However, because only the external behavior of the animal can be observed and the internal condition cannot be assessed, the animal’s state can easily be misjudged. Additionally, although some systems use heartbeat measurement to determine a state of tension, or use rest to assess the internal state, because an increase in heart rate can also occur as a result of exercise, it is desirable to use this measurement in combination with behavioral information. In the current study, we proposed a monitoring system for animals using video image analysis. The proposed system first extracts features related to behavioral information and the animal’s internal state via mask R-CNN using video images taken from the top of the cage. These features are used to detect typical daily activities and anomalous activities. This method produces an alert when the hamster behaves in an unusual way. In our experiment, the daily behavior of a hamster was measured and analyzed using the proposed system. The results showed that the features of the hamster’s behavior were successfully detected. When loud sounds were presented from outside the cage, the system was able to discriminate between the behavioral and internal changes of the hamster. In future research, we plan to improve the accuracy of the measurement of small movements and develop a more accurate system.

## 1. Introduction

In the current period of reduced social interaction because of the coronavirus disease 2019 (COVID-19), pet ownership may be helpful for improving quality of life. Pet ownership has been reported to have positive benefits, such as reducing psychological distress caused by loneliness [1] and improving health [2]. The pet population in the US is around 157 million, and the number of pet owners has increased in recent years because of increased income [3] and the greater proportion of time spent at home because of the COVID-19 pandemic. The number of pet owners is predicted to increase in the future [4,5] even after COVID-19. Pets are often thought of as members of the family, and it is desirable to be able to regularly monitor animals’ conditions considering the risk of injury or illness as well as their contribution to zoology.

Monitoring animals can be performed in various ways, such as by analyzing changes in behavior associated with changes in the living environment [6,7]. However, manual data collection can miss important information and has time constraints. Therefore, systematic and continuous monitoring is important [8]. In contrast, many monitoring methods using video cameras [9,10,11,12] and sensors such as thermography [13], as well as camera-based behavior analysis have been developed with recent improvements in deep neural network technology [14,15,16,17,18,19,20,21,22,23], making it possible to track animals with high accuracy in a non-contact manner. For example, for the care of orangutans in a zoo, a system used for human behavior analysis was adapted to perform individual identification, location tracking, and posture estimation using neural networks. These methods enable the detection of the locations in which each animal spends time in detail, and they can be applied to the optimal arrangement of the environment. However, it is not possible to determine animals’ internal state, which limits the understanding of the causes of their behavior. 

A previous study measured the heartbeat of animals to determine their internal state [24,25,26,27,28,29,30]. However, the effects of stress caused by the continuous wearing of sensors cannot be eliminated. In addition, various risks, such as contact infection, are associated with the use of sensors. Here, some systems perform heart rate detection by capturing color changes caused by blood flow from facial images taken by a video camera [25] or by measuring weak bodily movements caused by heartbeats with thermography [13]. Al-Naji et al. proposed the animal’s cardiopulmonary signal detection method from video images and achieved non-contact monitoring of animals in a zoo [26]. However, there are few systems that monitor the condition of animals by considering not only external behavioral information but also the internal state of each animal.

We previously developed a monitoring system that simultaneously performs behavior analysis via video image analysis and internal state extraction in a non-contact manner [31]. This system also detects behaviors that are not performed in everyday life, and which can detect conditions that differ from those in everyday life. However, the system requires specialized knowledge in the setting of threshold values and detection accuracy degradation because of objects in the environment.

Therefore, the current paper proposes a new monitoring system for pets that combines a new feature extraction method using deep neural network technology and an anomaly detection method. The proposed system simultaneously extracts behavioral and internal information from the detected region using a mask R-CNN. The system then automatically detects unusual behaviors using a one-class support vector machine (SVM), which is widely used for anomaly detection.

This paper is organized as follows: Section 2 presents the proposed monitoring system, data acquisition, and the experimental setup. The experimental results of the proposed system with a hamster are discussed in Section 3 and Section 4, respectively. Finally, Section 5 outlines a conclusion.

## 2. Materials and Methods

**Figure 1:**
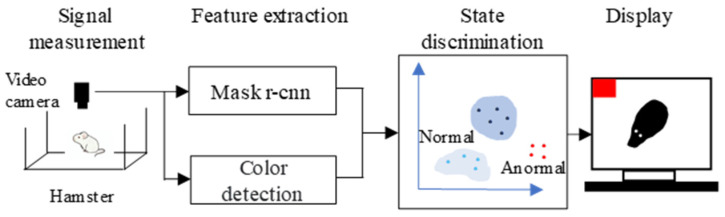
The structure of the proposed rodent monitoring system.

Figure 1 shows the proposed monitoring system. A digital hi-vision video camera (HC-W870M, Panasonic Corporation, Osaka, Japan) is used to analyze a hamster in a cage. Real-time monitoring can be performed using a PC with an HDMI capture device. When the system detects a state that is different from typical daily behavior, the system alerts the owner. The system consists of four parts of (1) signal measurement, (2) feature extraction, (3) state discrimination, and (4) display. The following subsections describe detailed information about each part.

### 2.1. Signal Measurement

A video camera is set up parallel to the ground without any tilt above a typical cage, and the height of the camera $T_{r}$ is adjusted to maximize the hamster’s view angle as much as possible to measure its daily behavior ( $T_{r}=0.4$ m). In this paper, the behavior of hamsters, including states such as the state of eating, was measured for approximately 3 min per day (2.8 ± 0.7 min, sampling frequency: $f_{s}=60$ Hz). A total of six days of video images were stored, and a clapping sound was generated at 90.7 s on one day of data collection.

**Figure 2:**
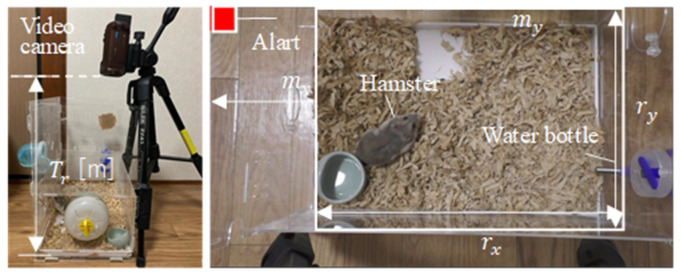
Overview of the proposed monitoring system.

**Figure 3:**
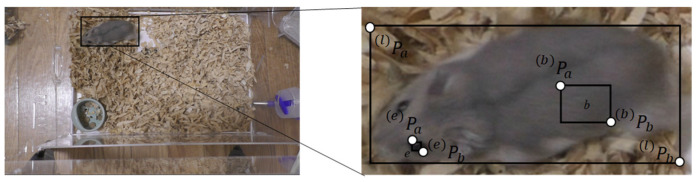
Regions for color information extraction.

From the video image measured by the video camera, a $r_{x}\times r_{y}=1370\times 880$ rectangular area A with margins $m_{x}=350$ and $m_{y}=0$ pixels in each axis direction is set from each frame to omit the living environment in the cage (see Figure 2). Here, the system first extracts the maximum length of the hamster from the image and color information to identify the hamster and the hamster’s eyes from the measured video image. The user clicks on the image of frame *t* that corresponds to each region, and the system automatically detects the minimum/maximum thresholds for hamster and hamster eye region detection: hthmax (b)=95 , hthmin (b)=60 , sthmin (b)=30 , sthmax (b)=0 , vthmax (b)=75 , vthmin (b)=125 ,  hthmax (e)=65 , hthmin=30 (e) , sthmax (e)=100 , sthmin (e)=50, vthmax=40 (e) , and vthmin (e)=10 (corresponding to each element in the HSV color system), and the body length $a_{x}=680$ and $a_{y}=150$ pixels of the hamster along the x/y axes are also determined in this paper. This process is performed for images in which the hamster is lying in the y - axis direction. Figure 3 shows an example. The user performs three processing steps on the image. The first is the extraction of color information. The user clicks diagonal points such as Pa  (b) and Pb  (b) in an area like *b* that contains the color of the hamster on the screen, and the color information in the rectangle is used as the hamster’s color information. In this case, we select a region that is as close to a single color as possible so that there is no variation in the color information of the region containing the hamster. Next, the color information of the hamster’s eyes is extracted. For the eye color information, Pa  (e) and Pa  (e) are specified to create a region that includes only the eyes, such as *e*. Finally, the body length of the hamster is extracted. The diagonal points Pa  (l) and Pb  (l) are specified so that the entire hamster is enclosed.

### 2.2. Feature Extraction

The proposed system uses both mask R-CNN and previously reported color information detection [31] to extract features related to posture and internal states. The behavioral information detects the amount of movement and the change in body and movement direction per frame, the internal state is obtained from the color information change from the surrounding pixels of the detected hamster area, and the heartbeat information is extracted.

#### 2.2.1. Behavioral Information 

First, the coordinates of the hamster’s center of mass at frame *t* are calculated from extracted information using mask R-CNN [32], which is capable of object detection and classification as well as object shape analogy. Mask R-CNN consists of three main components: the backbone, region proposal network (RPN), and head. The first part, the backbone, is a network consisting of multiple convolutional layers and is responsible for extracting features from the input image. A convolutional neural network (CNN) is a neural network with a convolutional layer and a pooling layer, which can be combined with a pooling layer to extract more specific information (e.g., the complex shape of an object) by deeply constructing the convolutional layer. Here, Inception ResNet v2 is used in this study. After this step, the RPN takes the features output from the CNN and selects candidate object-like regions. The features extracted by the CNN are sent to the box classification layer, which detects the likelihood that an object exists in the candidate region, and the box regression layer, which represents the deviation from the correct object region. The candidate regions are subject to deviation from the actual position in the original image due to compression by the CNN. The RoIAlign layer divides the candidate region into 3 × 3 feature maps. The values of each pixel point are interpolated by bilinear interpolation based on the four pixel points on the feature map, and these are interpolated by max or average pooling to obtain the reference points of the RoI region. Finally, classification, which determines what kind of object is in the candidate region obtained from the RPN, and region extraction, which detects the location of the object, can be used to extract what kind of object exists in what region of the image.

The mask image constructed using instance segmentation is obtained by inputting the image of region A to the model trained on the COCO dataset. Here, there are no hamsters (rodents) in the dataset, and it is usually difficult to classify the target from the mask image. Other than the hamster, the measurement environment (cage) contained only flooring materials and playground equipment. Thus, the mask images with animals are regarded as regions that include the hamster, and the largest of the masked regions is classified as the hamster region B. From the region B (instance mask) obtained here, the point set of the mask $\vec{x_{n}}=(M_{xi}\left(t\right),M_{yi}(t))(i=1,2,\ldots I)(n=1,2\ldots N)$ is extracted. To reduce processing, only the set of points $\vec{x_{i}}=(M_{xi}\left(t\right),M_{yi}(t))(i=1,2,\ldots I)(I<N)$ representing the hamster’s contour from the mask point $\left(M_{xi}\left(t\right),M_{yi}(t)\right)$ is extracted and the center of gravity ${\left[G_{x}\left(t\right),G_{y}\left(t\right)\right]}^{T}$ of the coordinates of the point set of the mask is calculated from $\vec{x_{i}}$ . Here, the following procedures were used to extract the amount of movement $m\left(t\right)$ , the body shape change $r^{\prime }\left(t\right)$ , and the change in direction $\theta^{\prime }\left(t\right)$ as behavioral information.

The amount of movement is calculated as the time difference of the extracted center of gravity according to the following equation:

(1) $$m\left(t\right)=||{\left[G_{x}\left(t\right),G_{y}\left(t\right)\right]}^{T}-{\left[G_{x}\left(t-1\right),G_{y}\left(t-1\right)\right]}^{T}||.$$

This extracts the degree to which the hamster is moving in the cage, and the larger the value, the greater the movement in the cage. 

The proposed system also extracts changes in the body shape and direction of movement as feature values for behavioral information. The body shape change $r^{\prime }\left(t\right)$ is calculated using the difference between the hamster’s center of gravity and eye position.

(2) $$r^{\prime }\left(t\right)=r\left(t\right)-r\left(t-1\right).\;r\left(t\right)=\sqrt{{\left(G_{x}\left(t\right)-F_{x}(t)\right)}^{2}+{\left(G_{y}\left(t\right)-F_{y}(t)\right)}^{2}}.$$

Here, ${\left[F_{x}\left(t\right),F_{y}\left(t\right)\right]}^{T}$ is the position of the hamster’s eyes extracted from the color information in the bounding box circumscribing area B [31]. If no target hamster is detected in the masked region by mask R-CNN, or if the masked region itself cannot be extracted at time *t*′, no information is extracted. Since $r\left(t\right)$ is the distance between the center of gravity and the head, a large value indicates that the hamster’s body is being stretched and a small value indicates that the hamster’s body is shrunken. Therefore, by calculating the time difference, $r^{\prime }\left(t\right)$ , we can extract the change in the hamster’s body shape per unit time.

**Figure 4:**
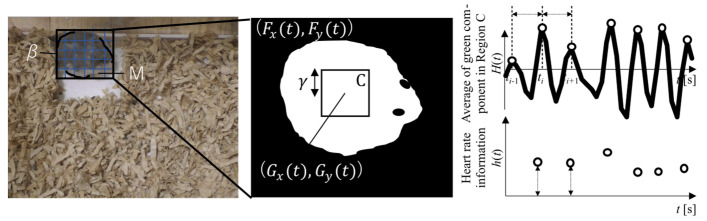
Region extraction based on mask R-CNN.

The change in movement direction is calculated from the direction of the hamster extracted by the following processes. Because the shape of a hamster is typically characterized by an elongated head as shown in Figure 4, the first principal component, from principal component analysis, and the vector indicating the direction of the hamster can be obtained. The set of point $\vec{x_{i}}$ representing the hamster’s contour is used to calculate the first principal component using the following equation:

(3) $$L\left(\lambda ,\;w\right)=w^{T}\Sigma w-\lambda \left(w^{T}w-1\right),$$

where Σ is the variance–covariance matrix of normalized $\vec{x_{i}}$ and $w={\left[w_{x},w_{y}\right]}^{T}$ is the eigenvector corresponding to the eigenvalue. The slope of the first principal component $w_{1}$ corresponding to the largest eigenvalue is obtained from Equation (3). Then, the following equation is used to extract the direction in the image where the *x*-axis positive direction is 0 rad:

(4) θt   (m)=tan−1(w1y/w1x). 

The direction θt   (c) calculated using a previously reported method [31] is also extracted as follows:

(5) θt   (c)=tan−1((Gyt−Fyt)/(Gxt−Fx(t))). 

The mean value $\theta \left(t\right)$ is obtained to extract information about the change in direction $\theta^{\prime }\left(t\right)$ as follows:

(6) θ′t=θt−θ(t−1)θt=θ   mt+θ   (c)t2

This allows for the evaluation of sudden movements, such as a hamster suddenly turning around and making a large change in its body direction. 

#### 2.2.2. Internal Body Information

Using a previously described method [31], a square region C of γ pixels around the hamster’s center of gravity was defined, and the average value $H\left(t\right)$ of the green component in region C was calculated (see Figure 4). The peak was detected from $H\left(t\right)$ after applying a second-order digital Butterworth filter with a cutoff frequency of ( $f_{l}=4.76$ , $f_{h}=7.14$ Hz). The mountain climbing method was applied to extract the peak-to-peak time interval *T*(*t*) of *H*(*t*).

(7) $$T\left(t\right)=t_{i}-t_{i-1}.\;$$

where $t_{i}$ is the time when the *i*-th peak of the detected *H*(*t*) appears. The inverse of the time difference was then multiplied by $t_{H}=0.7$ to detect the heart rate information $h\left(t\right)$ around $t_{H}$ seconds. 

Additionally, if there is no target hamster in the masked region, or if the masked region itself cannot be extracted, the center of gravity and eye position are calculated, using the respective coordinate values. Here, if the area is less than the area of $\alpha_{th}$ calculated from the maximum body length of the hamster set in advance, the heartbeat information is not extracted.

### 2.3. Anomaly Detection

On the basis of the extracted features, a one-class SVM is used to discriminate normal and anomalous activities of the hamster in daily life (Equation (8)).

(8) $$f\left(t\right)=w^{T}\emptyset \left(Z\left(t\right)\right)-p.$$

Given training data $Z^{q}={\left[m^{q},r^{q},\;{r^{\prime }}^{q},\;{\theta^{\prime }}^{q}{,\;h}^{q}\right]}^{T}$ (*q* = 1, …, *Q*), $Z^{q}$ is mapped to a high-dimensional feature space using a base function $\emptyset (Z^{q})$ and linear separation is performed in the mapped space. Here, anomaly values are mapped near the origin and normal values are mapped far from the origin in the feature space using a Gaussian kernel that represents the distance between the data. A hyperplane is then set up such that the margin for discriminating data groups near the origin from other data groups is maximized. Therefore, the parameters of discrimination function ***w*** and *p* (Equation (8)) are set such that Equation (9) is minimized so that ν data of the training data are near the origin.

(9) $$\frac{1}{2}{\left|\left|w\right|\right|}^{2}-p+\frac{1}{Q\nu }\sum_{j=1}^{Q}\xi_{j}.$$

where $\xi_{j}$ is a slack variable for soft margin maximization. After training, the newly extracted feature vectors can be input to the discriminant function (Equation (8)) to determine the state (e.g., normality is zero and anomality is one).

On the basis of the discrimination results, the user can judge the status of the hamster by displaying the results on the monitor. This system enables detailed monitoring of hamsters by using object detection as well as video image processing.

## 3. Experimental Results

To verify the effectiveness of the proposed system, we monitored the daily behavior of a hamster using the proposed system. We tried to reduce stress and prevent abnormal behavior caused by stress by making the environment the same in which the pets were actually kept and the measurement environment during the experiment. The environment is the same as the actual pet-keeping environment and a video camera is placed on top so that the movements of the hamsters can be observed (see Figure 2). In addition, to discriminate in the living environment, we conducted measurements every evening when nocturnal hamsters started their activities. 

As described in Section 2.1, the number of video images was six and a clapping sound was generated on one day of recording. The behavioral changes before and after the clapping sound were evaluated using the data (10,924 frames; 5062 samples were used to extract features). The data without sound generation were used as the training dataset (remaining 5 days; 48,255 frames; 23,179 samples were used to extract features). Five-fold cross-validation was used to determine the model parameters for which data without sound generation can be accurately discriminated as normal ( ν from 0.0001 to 0.01, γ [which is the parameter for the Gaussian kernel function to determine the decision boundary] from 0.01 to 1 in increments of 0.01). 

**Figure 5:**
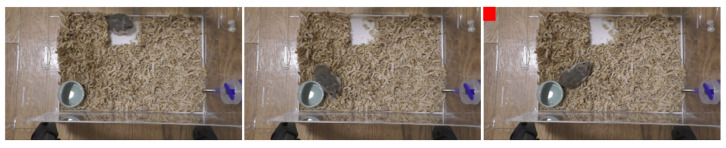
Scenes from the experiment.

**Figure 6:**
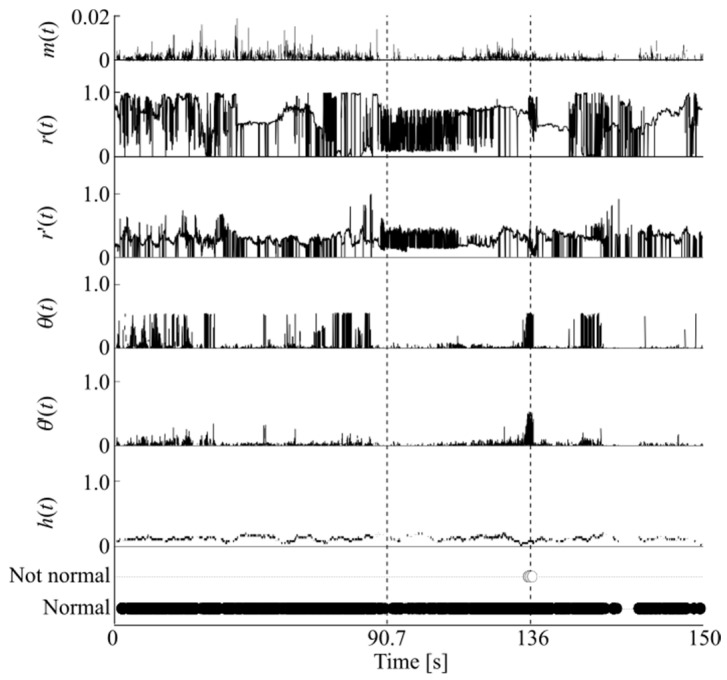
An example of experimental result.

Figure 5 shows scenes from the experiment and Figure 6 shows an example of the discrimination. Here, after the cross-validation, the highest discrimination rate was obtained when the parameters ν and γ were 0.0001 and 0.25, respectively, and the results were obtained using the parameters.

Table 1 shows the confusion matrix, with the results of two-class classifications using SVM, linear discriminant analysis (LDA), and artificial neural network (MLP: multi-layer perceptron with two hidden layers of twenty hidden units) for the data of the daily activity class (before sound generation, *f*(*t*) = 0) and the data for the entire time after sound occurrence as the non-daily activity class (*f*(*t*) = 1) for comparison. 

## 4. Discussion

From Figure 6, the discrimination was performed as normal before the sound was generated; however, at approximately 136 s after the sound was generated (90.7 s), the hamster’s state was judged as not normal. At this time, the hamster suddenly changes direction and its heart rate tends to decrease, indicating that the system is able to detect conditions different from normal daily activities simultaneously using behavioral information and internal states. Table 1 shows that SVM failed to discriminate the data well because all data were classified as the normal state while OCSVM can detect anomaly states. In both anomaly detection using OCSVM and when considering the two-class classification problem using SVM, the discrimination rate of non-daily activities did not have a high level of accuracy. This was mainly because the hamster shrank during the daily activities, and the direction vector was often inverted because of the noise caused by the hamster’s shadow. Additionally, because the training data also include infrequent situations in daily activities, it is possible that the discriminative boundary was not accurately determined. In fact, in the results of the other discrimination methods shown in Table 1, linear discriminant analysis hardly discriminated between normal and anomaly states. In addition, when an artificial neural network was used, it can be seen that misdiscrimination occurred in each of the normal and non-normal states. Two-class discrimination except for OCSVM does not discriminate correctly even though data from the non-normal state was also used for training.

**Figure 7:**
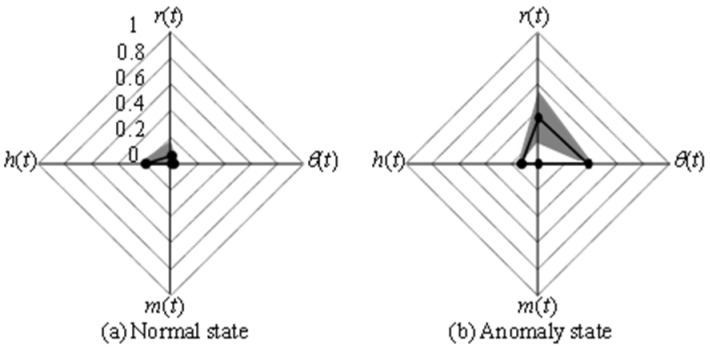
An examples of extracted feature vectors.

Figure 7 shows feature vectors extracted in (a) a normal state and (b) an anomaly state during the experiment. The solid line in the figure represents the mean value of each feature, and the shading represents the standard deviation. The figure shows that the variation in the behavioral information is large in all states. On the other hand, in the anomaly state, the value of the behavioral information is larger than in the normal state, and the value of *h*(*t*), which represents the internal state, appears to be smaller. In the continuous measurement of hamster behavior, hamsters were often observed to exhibit slight changes in response to the occurrence of abnormal sounds, but they continued their daily behavior for a short period of time afterward. Although such characteristics were often observed in general, when data that differed from the daily state that can be measured continuously were obtained using the anomaly detection method, it was possible to discriminate the anomalous state as an unusual state and to produce a warning. Therefore, the proposed method appeared to be extremely effective for extracting behaviors and internal states and identifying them via anomaly detection. 

These results indicate that the proposed system can detect conditions that are different from the everyday life of the animals by using just a video camera and this approach has been shown to be extremely useful in monitoring animals, including pets.

## 5. Conclusions

In this study, we proposed a monitoring system for hamsters using a single video camera. The system is capable of extracting different states from daily life using features of behavioral information, which determines the kind of movement performed by the animal, and internal information, which reflects the animal’s internal state. Thus, the system has enabled the user to analyze pets’ conditions without specialized knowledge. In an experiment, we investigated whether it was possible to detect behavioral changes in a hamster by stimulating it. In this experiment, a hamster’s daily life activity was measured using the proposed system, and video images containing routine behavior were stored. We also prepared videos of the hamsters being stimulated by clapping during the recording, and the changes in behavior before and after the generation of abnormal sounds caused by the clapping were evaluated. The results confirmed that the proposed system was able to discriminate daily activities before the clapping sound was generated with an accuracy of 100% by adjusting the parameters of the one-class SVM. This system also detected anomaly states after the clapping sound was generated. 

In the future, we aim to improve the discrimination accuracy by extracting more detailed behaviors, such as hand movements. The current results were obtained using only a few validation datasets with optimized parameters, and further validation with additional data is necessary. Furthermore, we plan to measure daily activities at night using sensors, such as a depth camera and thermography, so that the system can consider a wider variety of conditions.

## Figures and Tables

**Table 1:** Comparison results of state estimation.

	OCSVM		SVM		LDA		MLP	
	*f*(*t*) = 0	*f*(*t*) = 1	*f*(*t*) = 0	*f*(*t*) = 1	*f*(*t*) = 0	*f*(*t*) = 1	*f*(*t*) = 0	*f*(*t*) = 1
Normal	2674	0	2674	0	1145	1529	2667	7
Not normal	2377	11	2388	0	793	1595	2333	55

## Data Availability

Data available on request due to privacy and ethical restrictions.
